# Dual-Specific Interaction to Detect DNA on Gold Nanoparticles

**DOI:** 10.3390/s130505749

**Published:** 2013-05-03

**Authors:** Chuan-Liang Feng, Xiao-Qiu Dou, Qing-Lei Liu, Wang Zhang, Jia-Jun Gu, Shen-Min Zhu, Andrew Tobias Aveling Jenkins, Di Zhang

**Affiliations:** 1 State Key Lab of Metal Matrix Composites, School of Materials Science & Engineering, Shanghai Jiao Tong University, 800 Dongchuan Road, Shanghai 200240, China; E-Mails: douxiaoqiu@sjtu.edu.cn (X.-Q.D.); liuqinglei@sjtu.edu.cn (Q.-L.L.); wangzhang@sjtu.edu.cn (W.Z.); gujiajun@sjtu.edu.cn (J.-J.G.); smzhu@sjtu.edu.cn (S.-M.Z.); zhangdi@sjtu.edu.cn (D.Z.); 2 Department of Chemistry, University of Bath, Bath BA2 7AY, UK; E-Mail: a.t.a.jenkins@bath.ac.uk

**Keywords:** nanoparticles, fluorophore dye, quenching, streptavidin/biotin, DNA hybridization, sensors

## Abstract

An approach to selectively and efficiently detect single strand DNA is developed by using streptavidin coated gold nanoparticles (StAuNPs) as efficient quenchers. The central concept for the successful detection is the combination the of streptavidin-biotin interaction with specific probe-target DNA hybridization. Biotin labeled probe DNAs act as “bridges” to bring Cy5 labeled targets to the particle surface and the fluorophore dye can be rapidly and efficiently quenched by StAuPNs. By measuring the changes of photoluminescence intensity of Cy5, an efficient, selective, and reversed detection of DNA hybridization is realized. The methodology may pave a new way for simple and rapid detections of biomolecules.

## 1. Introduction

Due to their unique optical and electronic properties [1], DNA biosensors based on colloidal gold nanoparticles (AuNPs) show the merits of high sensitivity and selectivity to probe trace amounts of DNA targets, which can fulfil the stringent requirements of clinical genetic diagnosis, drug discovery, and the detection of environmentally hazardous compounds [2]. A biological or molecular coating acting as a bioactive and selective interface is often attached to the AuNPs prior to their application in biosensing systems. A target binding event (*i.e.*, DNA hybridization) occurring onto the AuNPs' surface may have a significant effect on their optical (change of the light absorption/emission) or electrochemical properties (oxidation/reduction current onto a transducing platform), which can offer novel alternatives for bioanalysis [3].

The interactions between AuNPs and the detected biomolecules can give rise to a variety of interesting phenomena, such as electro-mechanical detection, electrical and electrochemical detection, light absorption, light scattering, surface plasmon resonance, fluorescence quenching and so on [4]. In particular, AuNPs represent a new class of universal fluorescence quenchers that should find applications in molecular engineering and biosensor development [5]. In the case of DNA detection, probes DNA are often labeled with a thiol at one end and a dye at the other. Afterwards, the probes self-organize into a constrained conformation on the nanoparticle surface and the fluorophore dye is quenched by the particle [6]. Upon target binding, the constrained conformation opens and the fluorophore is separated from the particle surface, hence restoring luminescence. These phenomena qualify AuNPs as highly interesting substrates for rapid and sensitive detections of target oligonucleotides *via* fluorescence quenching between the particles and DNA molecules [7].

**Figure 1.**
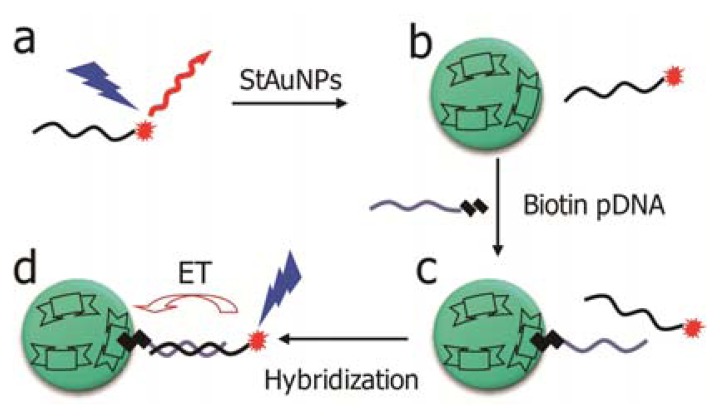
Schematic demonstration of DNA detection based on StAuNPs: (**a**) Cy5 labeled oligonucleotide targets (T1); (**b**) Mixture solution of T1 and StAuNPs; (**c**) The addition of biotin modified complementary biotin tagged probe oligonucleotide (pDNA); (**d**) With the hybridization, the energy transfer from Cy5 to AuNPs, resulting in subsequent energy quenching of Cy5.

This work will report a different approach to sensitively detect DNA hybridization using streptavidin coated AuNPs (StAuNPs) as efficient quenchers (Figure 1). The central idea for the successful detection is the combination of streptavidin-biotin interaction with specific probe-target DNA hybridization. Under the interactions, biotin labeled probe DNA act as “bridges” to bring Cy5 labeled targets to the particle surface and the fluorophore dye can be rapidly and efficiently quenched by StAuPNs. By measuring the changes of photoluminescence intensity of Cy5, an efficient, selective, and reversible detection of DNA hybridization is accomplished.

## 2. Experimental Section

*Materials:* Streptavidin coated gold nanoparticles were purchased from EY Laboratories Inc. (San Mateo, CA, USA) and used as received. Each AuNPs contains about five streptavidin units. Probe DNA and target DNA strands labeled with Cy5 were purchased from MWG-biotech AG (Ebersberg, Germany). The nucleotide sequence of the 30 mer probe DNA are: P1: 5'-biotin (TTT)_5_ TGT ACG TCA CAA CTA-3'; PNA3: 5'-(TTT)_5_ TGT ACG TGA CAA CTA-3'. The 15 mers with different mismatch used were: T1: 5'-Cy5-TAG TTG TGA CGT ACA-3' (MM0); T3: 5'-Cy5-TAG TTG TCA CGT ACA-3' (MM1); TMM: 5'-Cy5-TTT TTT TTT TTT TTT-3' (total mismatch).

*Detection of PL:* PL emission spectra were collected using a Tecan InfiniteM200 spectrometer (Männedorf, Switzerland). It records photoluminescence excitation and emission spectra with the emission range from 300 to 900 nm and excitation from 260 to 690 nm.

*DNA hybridization:* The assay solution was composed of Cy5 labeled DNA (10 nM), SAuNPs (1.2 nM) in 1 mL phosphate Buffer (pH 7.4). For investigating the DNA hybridization, different concentrations of probe DNA was prepared. Five μL probe solution with different concentrations was injected at room temperature inside the mixed solution each time to achieve different probe DNA concentration. The incubation time for each probe DNA is 20 min.

## 3. Results and Discussion

**Figure 2.**
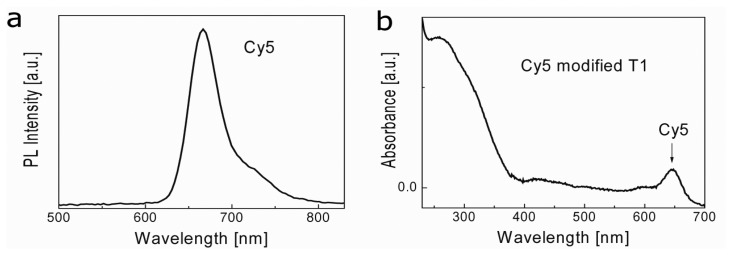
(**a**) PL emission spectra of Cy5 and (**b**) UV-Vis absorption spectra of Cy5.

To detect DNA hybridization, AuNPs coated with streptavidin (ca. 5 streptavidins/NP) were selected as fluorescence quenchers. Biotin tagged 30 mer oligonucleotide probe DNA (P1) and Cy5 labeled 15 mer oligonucleotide target (T1) DNA were used for studying hybridization. The photoluminescence (PL) and UV-vis spectra of Cy5 are shown in Figure 2.

**Figure 3.**
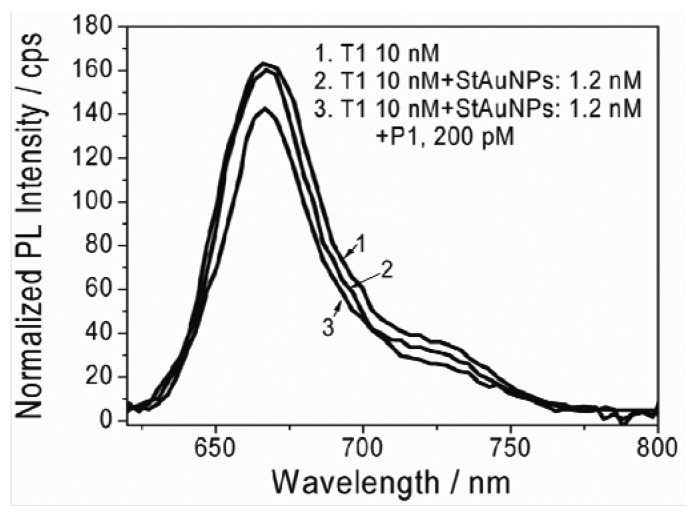
Changes in PL emission spectra of Cy5 after adding (**1**) T1; (**2**) 1.2 nM StAuNPs; (**3**) further 200 pM P1.

A solution with 10 nM T1 in PB buffer was made first and the distance separating two neighboring T1 molecules was about 1.5 μm, based on simple calculation (10 nmol T1 in 1 L solution). Upon adding StAuNPs (∼1.2 nM), a slight decrease of Cy5 emission intensity was observed (Figure 3), suggesting that there were slight interactions between StAuNPs and Cy5. Following the addition of P1 (200 pM, 5 μL PB solution), a distinct decrease of the PL intensity of Cy5 was observed because of the quenching of Cy5 by StAuNPs. The reason was that the P1 did not only interact with StAuNPs through avidin and biotin interaction, but also interacted with T1 through specific hybridization. Since the association constant between avidin and biotin is ∼10^15^ L/mol and the association constant between P1 and T1 is ∼10^9^ L/mol [8], it is highly plausible that most P1 probes are first adsorbed onto the StAuNPs surface and then interact with T1. Thus, P1 acted as a “bridge” and could bring T1 to the surface of StAuNPs via the specific interactions, which resulted in energy transfer (ET) from Cy5 to the StAuNPs and caused the decrease of PL intensity of Cy5 dyes.

**Figure 4.**
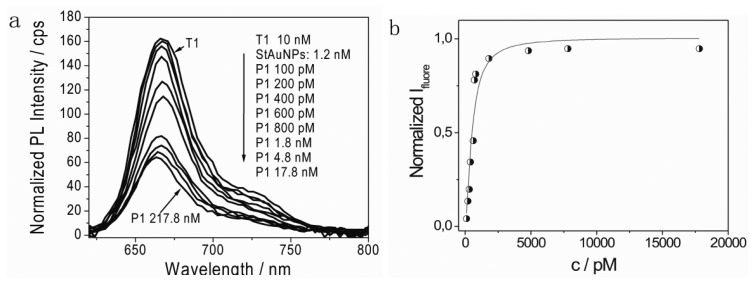
(**a**) Changes in PL emission spectra of Cy5 (T1) with varying P1 concentration from 100 pM to 17.8 nM. (**b**) The normalized fluorescence intensity *vs.* P1 concentration and the calculated KA (1.54 × 109 L·mol^−1^) for P1 hybridization with T1 from concentration at ½ I_fluoremaxa_.

A series study of the continuous changes of PL emission intensity with varying P1 concentration from 100 pM to 17.8 nM was followed [Figure 4(a)]. Firstly, a detection limit down to 100 pM was observed. The fluorescence intensity continuously decreased with further increasing P1 concentration. The maximum quenching efficiency of Cy5 was found at a P1 concentration of 17.8 nM and the calculated quenching efficiency was ∼60%.

**Figure 5.**
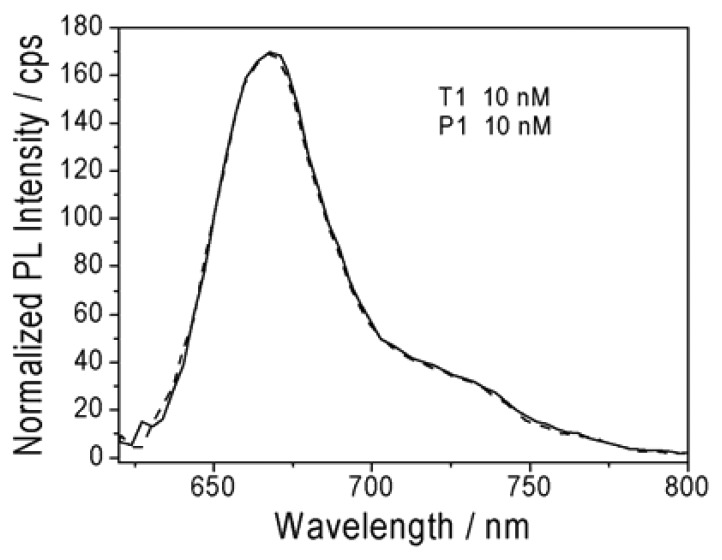
(**1**) PL emission spectra of Cy5. (**2**) Changes of PL emission spectra of solution after adding P1.

Considering the separation distance between Cy5 and StAuNPs here was about 18 nm (the diameter of a streptavidin is ∼5 nm [9] and the length of 30 base pair DNA is ∼13.04 nm in solution [10]), the result was in good agreement with the reported quenching efficiency (∼62%) [10]. A systematic study of the interaction between fluorophores and AuNPs under a similar separation distance between fluorophore and AuNPs has been investigated and revealed that AuNPs have a much longer interaction range over the fluorescence resonance energy transfer (FRET) distance [11]. This increases the probability of energy transfer and accounts for the enhanced efficiency of nanomaterial surface energy transfer (NSET) over FRET [12,13]. The calculated energy transfer distances are about 10 times greater than the typical Förster distance [14], which essentially extends the usable distances for measurement. Finally, as a control experiment, P1 (10 nM) was added in the solution with only T1 (10 nM) and no changes of PL intensity was detected (Figure 5), demonstrating that StAuNPs were necessary elements for the successful hybridization detection.

Since the association constant between streptavidin and biotin is much larger than the hybridization association constant between P1 and T1, we made an assumption that biotin labeled P1 preferred to first interact with StAuNPs and then hybridized with T1. Thus, the T1 should be in equilibrium with the ones bound to the sites of StAuNPs, any changes in the solution concentration of P1, *c*, hence resulted in the changes of surface coverage of hybridized complements (T1). By plotting these equilibrium intensities as a function of *c*, the Langmuir adsorption isotherm can be obtained according to:

(1) $$I_{fl}\left(c\right)\propto \Phi \left(c\right)=cK_{A}/\left(1+cK_{A}\right)$$

The calculated disassociation rate *K_D_* from concentration at ½ I_fluoremaxa_ was 6.5 × 10^−10^ mol/L [Figure 4(b)]. For Langmuir adsorption, the disassociation rate and association are reciprocal at equilibrium, that is, *K_A_* = 1/*K_D_*. Thus, the *K_A_* was calculated as 1.54 × 10^9^ L mol^−1^. This value is quite consistent with the hybridization constant between P1 and T1.

**Figure 6.**
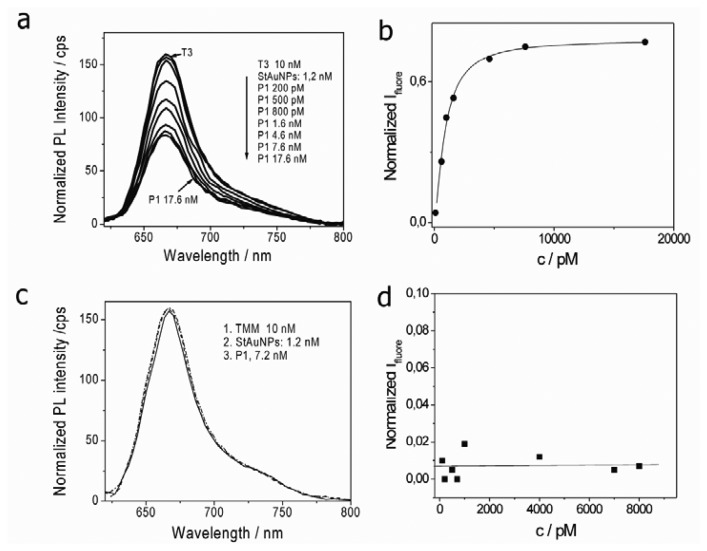
(**a**) Changes in PL emission spectra of Cy5 modified T3 with varying P1 concentration from 200 pM to 17.6 nM; (**b**) The normalized fluorescence intensity *vs.* P1 concentration and the calculated KA (7.8 × 10^7^ L·mol^−1^) from concentration at ½ I_fluoremaxa_ for hybridization with T3; (**c**) PL emission spectra of Cy5 modified TMM by adding 7.2 nM P1; (**d**) Fluorescence intensity of Cy5 modified TMM *vs.* P1 concentration.

To prove specificity of the hybridization, a series study of the continuous changes of PL emission intensity with varying P1 concentration from 200 pM to 17.6 nM were followed by using Cy5 labeled mismatch 1 DNA targets (T3) [Figure 6(a)]. The lowest detection limit was about 200 pM and the maximium PL quenching efficiency was ∼48% for T3. Based on Langmuir adsorption mode, the association rate *K_A_* for T3 was about 7.8 × 10^7^L·mol^−1^ [Figure 6(b)], in agree with the hybridization constant between DNA probes and mismatch 1 targets [15]. If Cy5 labeled completely mismatched DNA targets (TMM) were used, the PL intensity was decreased by ∼1% upon addition of P1 [Figure 6(c)]. Especially, PL intensity has no changes with P1 concentration as plotted in Figure 6(d). The different binding behaviors should be ascribed to the decrease of affinity constant between P1 and T3 and no affinity at all between P1 and TMM [16]. The results demonstrated that the system showed high selectivity during DNA hybridization detection.

**Figure 7.**
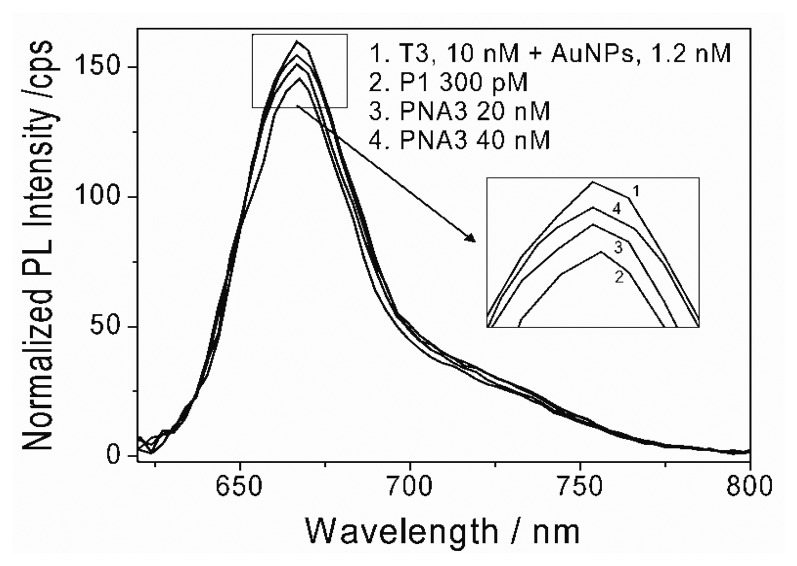
(**1**) PL emission spectra of T3 and StAuNPs mixture solution; Changes of PL emission spectra of solution after adding (**2**) 300 pM P1; (**3**) 20 nM PNA3; (**4**) 40 nM PNA3.

The reversed detection of DNA hybridization was also possible in the constructed system. To do this, non-biotin modified complementary DNA probes (PNA3) were selected. Biotin modified P1 (300 pM) was firstly added to the solution containing both StAuNPs and T3. A decrease of PL intensity was observed (Figure 7). The PNA3 with a concentration of 20 nM was then added to the solution. Surprisingly, a slow recovery of PL intensity was observed and PL intensity further increased with increasing PNA3 concentration up to 40 nM. This demonstrated that a reversible detection of DNA hybridization was possible in this process. Firstly, the selected PNA3 are not charged oligonucleotides and not modified by biotin either. This could avoid adsorption of PNA3 on the StAuNPs surface through streptavidin and biotin interaction, hence, PNA3 are freely dispersed in the solution. Secondly, since T3 were completely complementary with PNA3 but mismatch 1 with P1, there was a strong competition during hybridization between PNA3 and P1. Thus, T3 would prefer to re-hybridize with PNA3 after dissociation with P1 on the StAuNPs surface. As a result, the distance between T3 and StAuNPs surface increased again and quenching efficiency from Cy5 to StAuNPs decreased correspondingly, leading in turn to the increase of PL intensity. Here, excess PNA3 was used for obtaining better reversible binding. This experiment not only ruled out of the artificial influence during DNA hybridization but also proved that it was possible to achieve reversible hybridization detection in the StAuNPs-based system.

## 4. Conclusions

In summary, a new simple method based on AuNP suspensions has been developed to detect DNA hybridization with a highly selective and sensitive system. The successful detection is achieved by utilizing the combination of streptavidin/biotin interaction with DNA probes/targets hybridization as well as the AuNP fluorophore quenching effect. By measuring the quenching of different fluorophores, multiple probe DNA detection becomes possible in the StAuNPs-based nanostructures if DNA targets labeled with different fluorophores (with different emission wavelengths) are mixed in the same solution. The described methodology could be extended to a high throughput format and potentially become a new generation nanotechnology for biodiagnostics. In addition, the experiment can be designed to bring Cy5 much closer to Au surface, e.g., if Cy5 is labeled at the other end of targets compared with the ones used in this experiment, Cy5 dyes can be brought much closer to the gold surface and the quenching efficiency will be much higher. These experiments are now in progress in our research group.

## References

[1] Katz E., Willner I. (2004). Integrated nanoparticle-biomolecule hybrid systems: Synthesis, properties, and applications. Angew. Chem. Int. Ed..

[2] Lu N., Pei H., Ge Z.L., Simmons C.R., Yan H., Fan C.H. (2012). Charge transport within a three-dimensional DNA nanostructure framework. J. Am. Chem. Soc..

[3] Niemeyer C.M. (2001). Nanoparticles, proteins, and nucleic acids: Biotechnology meets materials science. Angew. Chem. Int. Ed..

[4] Moirangthem R.S., Chang Y.C., Wei P.K. (2011). Investigation of surface plasmon biosensing using gold nanoparticles enhanced ellipsometry. Opt. Lett..

[5] Liu M., Zhao H.M., Chen S., Yu H.T., Quan X. (2012). Interface engineering catalytic graphene for smart colorimetric biosensing. ACS Nano.

[6] Rosi N.L., Mirkin C.A. (2005). Nanostructures in biodiagnostics. Chem. Rev..

[7] Yeh H.C., Sharma J., Han J.J., Martinez J.S., Werner J.H. (2010). A DNA-Silver nanocluster probe that fluoresces upon hybridization. Nano Lett..

[8] Cady N.C., Strickland A.D., Batt C.A. (2007). Optimized linkage and quenching strategies for quantum dot molecular beacons. Mol. Cell. Probes.

[9] Hytönen V.P., Määttä J.A.E., Kidron H., Halling K.K., Hörhä J., Kulomaa T., Nyholm T.K.M., Johnson M.S., Salminen T.A., Kulomaa M.S. (2005). Avidin related protein 2 shows unique structural and functional features among the avidin protein family. BMC Biotech..

[10] Ray P.C., Fortner A., Darbha G.K. (2006). Gold nanoparticle based FRET asssay for the detection of DNA cleavage. J. Phys. Chem. B.

[11] Zhang W., Govorov A.O., Bryant G.W. (2006). Semiconductor-metal nanoparticle molecules: Hybrid excitons and the nonlinear Fano effect. Phys. Rev. Lett..

[12] Yun C.S., Javier A., Jennings T., Fisher M., Hira S., Peterson S., Hopkins S., Reich N.O., Strouse G.F. (2005). Nanometal surface energy transfer in optical rulers, breaking the FRET barrier. J. Am. Chem. Soc..

[13] Ray P.C., Darbha G.K., Ray A., Walker J., Hardy W. (2007). Gold nanoparticle based FRET for DNA detection. Plasmonics.

[14] Föster T. (1948). Zwischenmolekulare Energiewanderung und Fluoreszenz. Ann. Phys..

[15] Chu L.Q., Knoll W., Foerch R. (2006). Biologically multifunctional surfaces using plasma polymerization methods. Surf. Plasm. Poly..

[16] Liebermann T., Knoll W., Sluka P., Herrmann R. (2000). Complement hybridization from solution to surface-attached probe-oligonucleotides observed by surface-plasmon-field-eahanced fluorescence spectroscopy. Colloid Surf. A.

